# A New Local Anesthetic

**Published:** 1898-03

**Authors:** 


### SELECTIONS.

### A New Local Anesthetic.

A. Einhorn and Heinz(*Munch. Med. Woch.*) describe the new synthetic local anesthetic, orthoform. This body is a white, light powder, without smell or taste. It is only partially soluble in water, but enough is brought in solution to make the fluid anesthetic. It combines with hydrochloric acid, forming a very soluble compound, which cannot, however, always be used, as it irritates some mucous membranes, such as the conjunctiva. Anesthesia is only induced in the places with which the orthoform comes into contact. Orthoform acts as an anesthetic wherever it comes into contact with the nerves and thus it has no effect when applied to the unbroken skin. If it be applied to a burn of the third degree, the anesthetic effect is remarkable. It also allays the pain of ulcers, whether cancerous or other. In one case as much as fifty grammes was sprinkled on a wound within a week, showing that it is quite harmless. It is strongly disinfectant, hindering decomposition and fermentation. Orthoform was also useful in ulceration of the larynx; after some of the powder was blown in the pain was relieved for twenty-four hours. In gastric ulcer and carcinoma it was also of service, but much less so in chronic gastric catarrh. For external use the free orthoform is the best, but for internal use the soluble acid salt. Further observation is needed in regard to its action on the mucous membranes of the mouth, nose and naso-pharynx. As it is non-poisonous, it can be applied to large ulcerating surfaces. Internally, one-half to nineteen of the hydrochlorate has been given several times in the day. Orthoform is stable, non-hygroscopic, and can be added to other remedies.—*Indian Lancet.*

SULPHUR, even in such a minute quantity as the fraction of a grain, will increase the efficacy of a purgative pill or powder, by increasing the flatus in the intestine, thus facilitating the expulsion of its contents.

